# Poor Nutritional Status and Dynapenia Are Highly Prevalent in Post-Acute COVID-19

**DOI:** 10.3389/fnut.2022.888485

**Published:** 2022-06-03

**Authors:** Francesco de Blasio, Luca Scalfi, Bianca Castellucci, Anna Maria Sacco, Giulia Miracco Berlingieri, Ludovica Capitelli, Paola Alicante, Alessandro Sanduzzi, Marialuisa Bocchino

**Affiliations:** ^1^Respiratory Medicine and Pulmonary Rehabilitation, Clinic Center, Private Hospital, Naples, Italy; ^2^Department of Public Health, Federico II University, Naples, Italy; ^3^Respiratory Medicine Unit, Department of Clinical Medicine and Surgery, Federico II University, Naples, Italy

**Keywords:** post-acute COVID-19, malnutrition, phage angle, handgrip strength, dynapenia

## Abstract

Poor nutritional status is common (estimated prevalence 5–69%) in acute coronavirus disease-2019 (COVID-19), and has been associated with hospitalization, the need for intensive care, and mortality. Body composition (BC) and muscle function have also been related in such patients to poor disease outcomes.

As the evidence in the literature is limited, a cross-sectional study was carried out to determine the frequency of malnutrition in a cohort of post-acute COVID-19 patients referred to a rehabilitation center after hospital discharge. BC and muscle strength were assessed and the differences between bedridden and not bedridden patients were specifically evaluated.

The study sample was composed of 144 post-acute COVID-19 patients (mean age 64.8 years; males = 95), 37% of whom were bedridden (males = 60%). Nutritional status was evaluated with Mini-Nutritional Assessment (MNA) and Controlling Nutritional status (CONUT). Fat-free mass (FFM) and skeletal muscle mass (SM) were estimated using bioelectrical impedance analysis (BIA). Raw BIA variables (phase angle = PhA and impedance ratios = IRs) were also determined and handgrip strength (HGS) was measured. Dynapenia was identified according to the 2019 EWGSOP criteria.

According to MNA, 18% (*n*. 26) of patients were malnourished and 62% (*n*. 89) were at risk of malnutrition. As for CONUT, 21% (*n*. 31) of cases had moderate–severe malnutrition and 58% (*n*. 83) had light malnutrition. Abnormalities of raw BIA variables (low PhA and high IRs) and low HGS were more common in bedridden patients, in those who were malnourished, or had low FFM or SM. Dynapenic patients were 65% men and 47% women.

In conclusion, malnutrition, BC alterations, and low HGS occur in post-acute COVID-19 patients and are more common in bedridden patients. Further studies are needed to identify reliable algorithms for assessing nutritional status in post-acute COVID-19 patients undergoing rehabilitation.

## Introduction

Coronavirus disease-2019 (COVID-19) is a multi-organ disease primarily affecting the lung, due to acute infection by severe acute respiratory syndrome-Coronavirus-2 (SARS-CoV-2). *Sequelae* of COVID-19 are observed in a high proportion of patients in all the body organ systems both in the short (post-acute) and long term (the so-called long-COVID-19 syndrome) (1).

The poor nutritional status of COVID-19 patients may be due to multiple factors, such as inflammation, hyper-catabolism, increased work of respiratory muscles, metabolic/endocrine disorders, and specific therapies (2–4). Using different tools and criteria for screening/diagnosis, the prevalence of malnutrition has been estimated between 5 and 69%, and the risk of malnutrition between 39 and 86% (2). From a clinical point of view, malnutrition has been associated with negative outcomes, for instance, prolonged hospitalization (5), hospital deaths, and intensive care unit (ICU) admission (6).

The evaluation of body composition (BC) and muscle function plays a major role in the assessment of nutritional status. Considering the clinical setting, bioelectrical impedance analysis (BIA) is a non-invasive and widely used method, already applied in COVID-19 patients, which gives estimates of fat-free mass (FFM) and other body compartments such as skeletal muscle mass (SM) (7, 8). In addition, directly measured raw BIA variables (phase angle = PhA and impedance ratio = IR) yield information on inherent characteristics of FFM or muscle mass that may be related to body cell mass (BCM) and the ratio between extracellular water and total body water (ECW/TBW). Actually, low PhA has been observed in different categories of hospitalized COVID-19 patients and related to disease severity, length of stay, and mortality (9–11), with little evidence available in post-acute COVID-19 (8, 12).

As far as muscle function is concerned, low handgrip strength (HGS), a proxy marker of muscle strength and a marker of sarcopenia, has been associated with all-cause and disease-specific mortality, future function, bone mineral density, fractures, cognition and depression, and hospitalization (13). In critical ICU COVID-19 patients, muscle wasting and decreased muscle strength occurred early and rapidly (14) and still persisted at 3 months from discharge (15); similar observations were also reported in patients who recovered from mild-to-moderate disease (16). Thus, in COVID-19 patients, muscle weakness, fatigue, and low exercise capacity may be related to poor muscle quality and low HGS (17), while a lower HGS is an independent risk factor for disease severity (18, 19), longer hospitalization (20), and mortality (21).

Due to the paucity of data concerning nutritional status in post-acute COVID-19, the aim of this cross-sectional study was to assess the frequency of malnutrition, evaluated by different tools and criteria, in a cohort of post-acute COVID-19 patients referred to a rehabilitation center after hospital discharge. Nutritional status was determined using Mini-Nutritional Assessment (MNA) and Controlling Nutritional status (CONUT), together with BIA-derived BC, raw BIA variables, and HGS. Finally, the differences between bedridden and not bedridden patients were analyzed.

## Materials and Methods

### Study Population

The study population was composed of 144 consecutive clinically stable patients affected by COVID-19-related pneumonia discharged from hospital wards and admitted, from January 2021 to May 2021, to the Pulmonary Rehabilitation Unit–Clinic Center-Napoli, Italy. Inclusion criteria were as follows: age >18 years; the previous infection by SARS-CoV-2 according to the positive result on a reverse transcriptase polymerase chain reaction (RT-PCR) assay on nasopharyngeal swab; radiological evidence of pneumonia during the previous hospitalization; two consecutive RT-PCR SARS-CoV-2 negative results at hospital discharge. Exclusion criteria were osteo-muscular and neurological disorders or the presence of a pacemaker/implantable cardioverter defibrillator. Demographics, main clinical data, and laboratory parameters of interest for the study were anonymously collected into a dedicated database. The study was approved by the local Institutional Ethics Committee (protocol number AOC/0022330/2021) and performed according to the amended Declaration of Helsinki. All patients gave their written informed consent.

### Anthropometry and Body Composition

Anthropometric and BIA measurements were performed within 30 h after admission. Bodyweight and stature were measured to the nearest 0.1 kg and 0.1 cm with a mechanical column scale and a stadiometer, respectively (SECA 711 and SECA 220, Hamburg, Germany). In bedridden patients, supine height was determined with a modified stadiometer (SECA 213) and weight with a multifunctional/invalid chair scale (Soehnle 7708). The body mass index (BMI) was calculated as body weight (kg) divided by stature squared (m^2^).

Bioelectrical impedance analysis measurements were carried out on both body sides with a Human Im-Touch analyzer (DS Medica S.r.l., Milan, Italy) in standardized conditions (i.e., ambient temperature 23–25°C, fast >4 h, empty bladder, supine position for at least 10 min before testing). After cleaning the skin surface, the patients laid down with their legs and arms slightly abducted to avoid any contact between the limbs and the trunk. A standard tetra-polar technique was used: measuring electrodes were placed on the wrist and ankle dorsal surface, while injecting electrodes were on the dorsal surface of the hand and the foot, respectively. Impedance (*Z*) was measured at 5–50 to 100–200 kHz and PhA at 50 kHz by injecting an alternating current of 800 mA. IRs were defined as the ratio between Z at high frequency and *Z* at low frequency; two IRs were derived between *Z* at 100 or 200 kHz and *Z* at 5 kHz (IR100/5 and IR 200/5, respectively). The values for IRs and PhA were the mean of those obtained for the dominant (D) and non-dominant (ND) body side.

Fat-free mass and SM were estimated using the predictive equations proposed by Rutten et al. and by Jenssen et al., respectively (22, 23). FFM index (FFMI) was calculated as FFM/stature^2^ and SM index (SMI) as SM/stature^2^, while fat mass (FM) was obtained by subtracting FFM from body weight. Low FFMI values were those <17 kg/m^2^ for men and <15 kg/m^2^ for women (24).

### Handgrip Strength and Dynapenia

Handgrip strength was measured by the same operator following standard procedures (25) using a Dynex dynamometer (MD systems Inc., Ohio, USA) to assess the isometric strength of the upper limb. Three measurements were performed in the standing position for each hand, 1 min apart, with the elbow straight and fully extended, alternating between D and ND sides. The maximum value was derived for each arm, while HGS was defined as the highest value of the six attempts and also compared to local reference values (see above). Bedridden patients unable to stand unassisted were asked to perform the test in the seated position in a wheelchair. Patients were sitting straight up on the bench with arms at the side and both feet placed on the ground. Dynapenia was defined according to the 2019 EWGSOP criteria: <27 kg in men and <16 kg in women (13).

### Risk of Malnutrition and Malnutrition Assessment

Nutritional status was assessed with two different tools, MNA and CONUT. As for MNA, a score <17 identified malnourished patients, a score of 17–23.5 for those at risk of malnutrition, and a score ≥24 for those with a normal nutritional status (26). The CONUT score was based on lymphocyte count, total cholesterol, and serum albumin (27); a score of 0–1 was considered normal, whereas scores of 2–4, 5–8, and 9–12 were suggestive of light, moderate, and severe malnutrition, respectively. Laboratory parameters of interest for the study including the absolute number/mm^3^ of peripheral lymphocytes and serum levels of total cholesterol, albumin, and C-reactive protein (CRP) were retrieved from routine blood tests performed upon admission to the Pulmonary Rehabilitation Unit.

### Statistical Analysis

Results are expressed, where appropriate, as mean and standard deviation (SD), median value and interquartile range (*IQR*), minimum and maximum values, or frequency. The standard error (*SE*) was used in reporting data after controlling for confounders. ANOVA with the *post hoc* Tukey test and the general linear model (GLM) were used to compare groups and assess the effects of factors on a single dependent variable (also after adjusting for covariates). The chi-squared test was used to assess differences in frequencies between groups. The partial correlation was used to identify predictors of a given dependent variable. Local reference values (percentiles of interest) for raw BIA variables and HGS were derived in 210 male and 234 female healthy volunteers (age 20–40 years, BMI 20–29 kg/m^2^). The statistical significance was pre-determined as *p* < 0.05. All statistical analyses were performed with the Statistical Package for Social Sciences (SPSS Inc. Chicago, IL, USA) version 26.

## Results

### Demographics and Clinical Characteristics of the Study Population

The main demographics and clinical features of patients are reported in Table 1. Age ranged between 45 and 85 years (105 patients [73%] ≥60 years) with a median value of 65 years. The overall prevalence of underweight, overweight, and obese patients was 4% (*n*. 6), 32% (*n*. 46), and 28% (*n*. 40), respectively. A total of 80% of the study sample had at least one comorbidity, with systemic arterial hypertension and type II diabetes being the most prevalent (Table 1), while 37% of the patients were bedridden (60% males).

**Table 1:** Demographic and clinical characteristics of the study population.

	**Patients**		
	**Total**	**Males**	**Females**
	**(*n* = 144)**	**(*n* = 95)**	**(*n* = 49)**
Age (yrs)	64.8 ± 10.7	64.3 ± 10.7	65.6 ± 10.8
Stature (cm)	167.6 ± 9.6	171.9 ± 8.0	159.1 ± 6.3^*^
Weight (kg)	80.7 ± 17.6	82.8 ± 17.8	76.6 ± 16.6^**^
Body mass index (kg/m^2^)	28.7 ± 5.8	27.9 ± 5.1	30.3 ± 6.7^**^
Weight status			
underweight (BMI < 21 kg/m^2^)	6 (4)	6 (6)	0
normal weight (BMI >21 and <25 kg/m^2^)	52 (36)	29 (30)	23 (47)
overweight (BMI >25 and <30 kg/m^2^)	46 (32)	36 (38)	10 (20)
obese (BMI ≥30 kg/m^2^)	40 (28)	24 (25)	16 (33)
Smoking status			
current smokers	64 (44)	34 (36)	30 (61)
former smokers	13 (9)	12 (13)	1 (2)
no smokers	67 (46)	49 (52)	18 (37)
Comorbidities	115 (79)	74 (78)	41 (84)
Type II diabetes	35 (24)	27 (28)	8 (16)
Systemic arterial hypertension	89 (62)	55 (58)	34 (69)
Cardiovascular diseases	28 (19)	18 (19)	10 (2)
Chronic renal failure	9 (6)	6 (6)	3 (6)
Thyroid disease	14 (10)	7 (7)	7 (14)
Bronchial asthma	13 (9)	5 (5)	8 (16)
Bedridden	53 (37)	32 (34)	21 (43)

*Data are expressed as mean ± standard deviation (SD), or number of patients and (percentage)*.

* *p < 0.001*;

** *p < 0.05*.

*BMI, body mass index*.

The median length of the previous hospitalization was 27 days (*IQR* = 18–39), with no sex differences. In total, 53 patients (37%) came from respiratory sub-intensive care units and the remaining ones from hospital wards. Patients were directly referred from the hospital to the rehabilitation unit within 2 days after two consecutive RT-PCR SARS-CoV-2 negative results.

During the previous hospitalization, all patients had been treated with systemic corticosteroids. On admission, 68% of patients were still receiving low-dose oral steroids. In half of the cases, serum CRP was above 5 mg/L. Continuous oxygen supplementation was still required in 38% of cases.

### Malnutrition Is Highly Prevalent in Post-Acute COVID-19

**Figure 1:**
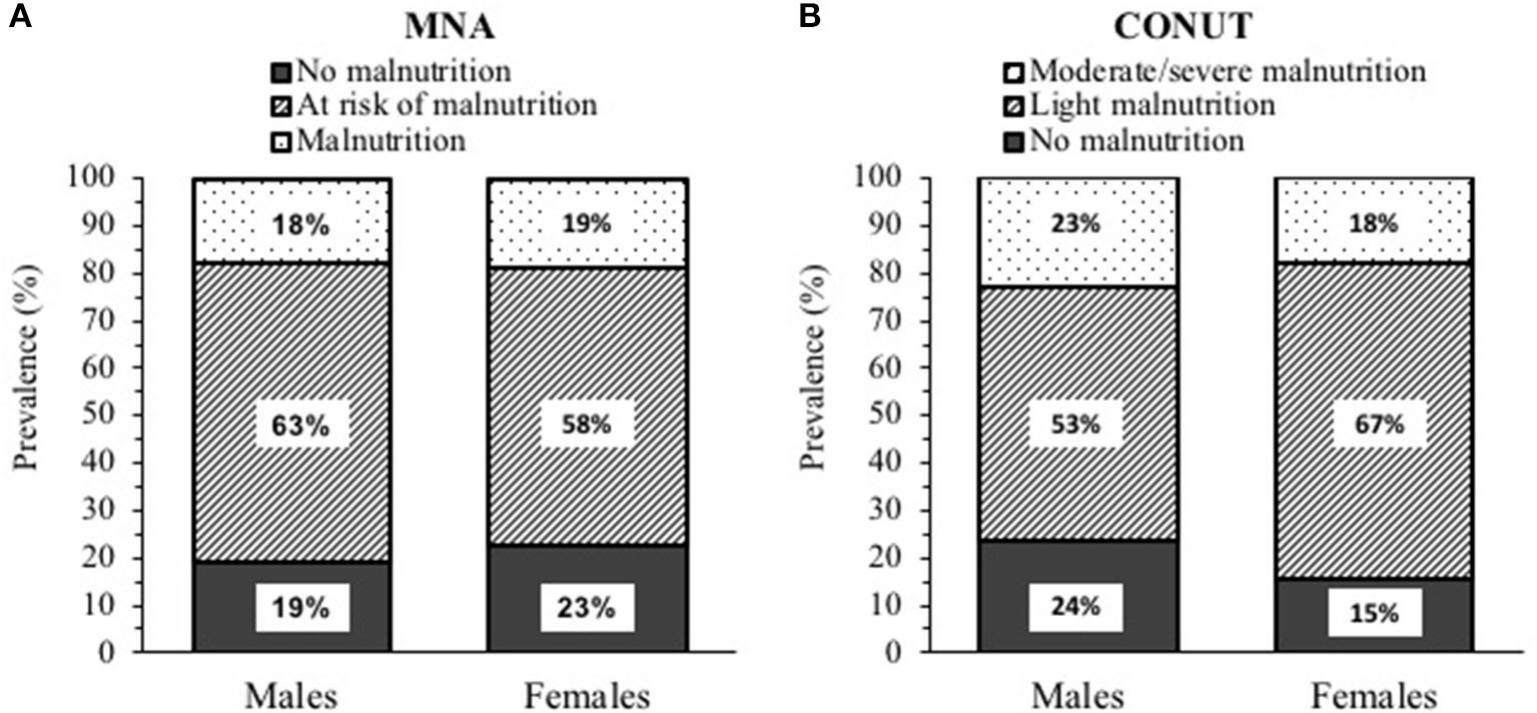
Prevalence of malnutrition in post-acute Coronavirus 2019 (COVID-19) patients (95 males and 49 females) according to: **(A)** Mini Nutritional Assessment (MNA) and **(B)** Controlling nutritional status (CONUT).

According to the MNA score (20.4 ± 3.8 in men and 20.4 ± 4.0 in women), 18% (*n*. 26) of patients were malnourished and 62% (*n*. 89) were at risk of malnutrition (Figure 1A). When CONUT was applied, 21% (*n*. 31) of patients showed moderate–severe malnutrition, and 58% (*n*. 83) had light malnutrition (Figure 1B) with regard to the components of the CONUT, low serum albumin was found in 57% (*n*. 82), low total serum cholesterol in 71% (*n*. 102), and low blood absolute lymphocyte count in 30% (*n*. 43) of the study sample.

### Phase Angle and IRs Are Severely Compromised in Post-Acute COVID-19

As shown in Table 2, female patients had lower FFM (−21%), FFMI (−7%), SM (−16%), and SMI (−20%) compared to men, but higher body fat percentage (+25%). Low values were observed in 17% (*n*. 24) and 30% (*n*. 43) of the whole sample for FFMI and SMI, respectively, being more common in the malnourished patients as identified by MNA (36% and 56%).

**Table 2:** Body composition and handgrip strength in post-acute COVID-19 patients.

	**Patients**		
	**Total**	**Males**	**Females**
	**(*n* = 144)**	**(*n* = 95)**	**(*n* = 49)**
Body composition			
Fat-free mass (kg)	52.3 ± 9.3	55.7 ± 8.0	44.1 ± 6.7^*^
Skeletal mass (kg)	23.3 ± 6.0	26.1 ± 4.8	17.7 ± 3.7^*^
Body fat percentage (%)	34.8 ± 8.2	31.8 ± 6.9	42.1 ± 6.2^*^
Fat-free mass index (kg/m^2^)	18.5 ± 2.1	18.9 ± 1.9	17.6 ± 2.5^*^
Skeletal mass index (kg/m^2^)	8.2 ± 1.6	8.8 ± 1.3	7.0 ± 1.4^*^
Raw BIA variables			
PhA at 50 kHz (degrees)	3.92 ± 1.12	4.10 ± 1.13	3.58 ± 1.03^*^
IR Z 100 kHz/Z 5 kHz	0.878 ± 0.034	0.871 ± 0.034	0.890 ± 0.029^*^
IR Z 200 kHz/Z 5 kHz	0.821 ± 0.040	0.814 ± 0.040	0.835 ± 0.035^*^
Handgrip strength	23.3 ± 9.8	27.2 ± 8.9	15.8 ± 6.4^*^
Dominant side	22.6 ± 10.0	26.6 ± 9.1	14.9 ± 6.2^*^
Non-dominant side	21.6 ± 9.4	25.0 ± 8.9	15.0 ± 6.5^*^

*Data are expressed as mean ± standard deviation (SD)*.

* *p < 0.001*.

*COVID-19, coronavirus disease-2019; PhA, phase angle; IR, impedance ratio; handgrip strength, maximum value considering both arms*.

Concerning raw BIA variables (Table 2), PhA varied from 1.50 to 6.35 degrees (min/max) and IR200/5 from 0.757 to 0.919. PhA50 (−0.047 degree/year) and IR200/5 (+0.002/year) changed with age (*p* < 0.001) but similarly in both sexes. When compared to local reference data, values of PhA below the 1st percentile were observed in 92% of male and 80% of female patients, while those of IR200/5 above the 99th percentile were found in 50% and 53%, respectively.

After adjusting for sex and age, raw BIA variables differed (*p* < 0.001) in patients with low SMI compared to the remaining ones, with PhA being 3.35 ± 0.01 *vs*. 4.17 ± 0.01 degrees and IR200/5 0.852 ± 0.005 *vs*. 0.829 ± 0.003, respectively (mean ± SE, after controlling for sex and age). In addition, as shown in the Supplementary Material (Supplementary Table 1), PhA was markedly lower and IRs greater in malnourished patients as identified by either MNA or CONUT.

### Dynapenia Is Highly Prevalent in Post-Acute COVID-19

Handgrip strength was greater by 72% in males (min 8.6 kg, max 46.4 kg) than in females (min 5.6 kg, max 29.8 kg) post-acute COVID-19 patients (Table 2), and the difference persisted even after adjustment for age and FFM (data not shown). According to the EWGSOP 2019 criteria, dynapenic patients were 65% (*n*. 62) in men and 47% (*n*. 23) in women. In addition, 76% (*n*. 110) of male and 60% (*n*. 29) of female patients had HGS below the 1st percentile of local reference values. HGS was lower in patients with low FFMI or SMI, and also in malnourished patients (according to MNA but not to CONUT) by 37% compared to the well-nourished patients (data not shown).

**Figure 2:**
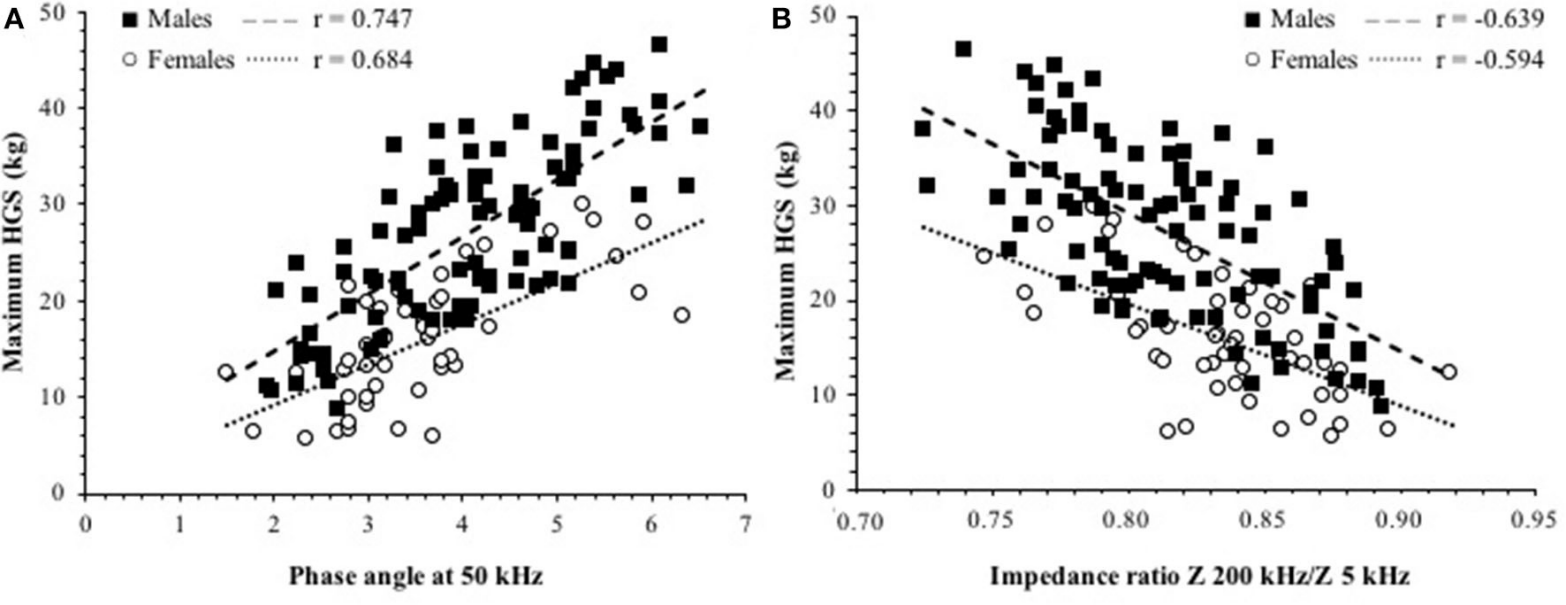
Relationship between maximum HGS and **(A)** phase angle (PhA) at 50 kHz. **(B)** IR Z 200/Z 5 kHz in patients. The *p* < 0.001 for all the correlation coefficients. The intercepts were significantly different between sexes in both cases.

In bivariate analysis, after adjusting for sex, HGS correlated (*p* < 0.001) with PhA (*r* = 0.727), IR100/5 (*r* = −0.654), and IR200/5 (*r* = −0.626), while a less strong association was observed with FFM (*r* = 0.386) and SM (*r* = 0.502) and a weak correlation with weight (*r* = 0.278), BMI (*r* = 0.197), and with CRP (*r* = −0.181). The relationships of HGS with PhA or IR200/5 in either sex are shown in Figure 2. In addition, HGS is also correlated with MNA score (*r* = 0.502, *p* > 0.001) and CONUT score (*r* = −0.217, *p* < 0.05).

### Higher Malnutrition and More Pronounced Alterations of Raw BIA Variables and Handgrip Strength Occur in Bedridden Patients

**Figure 3:**
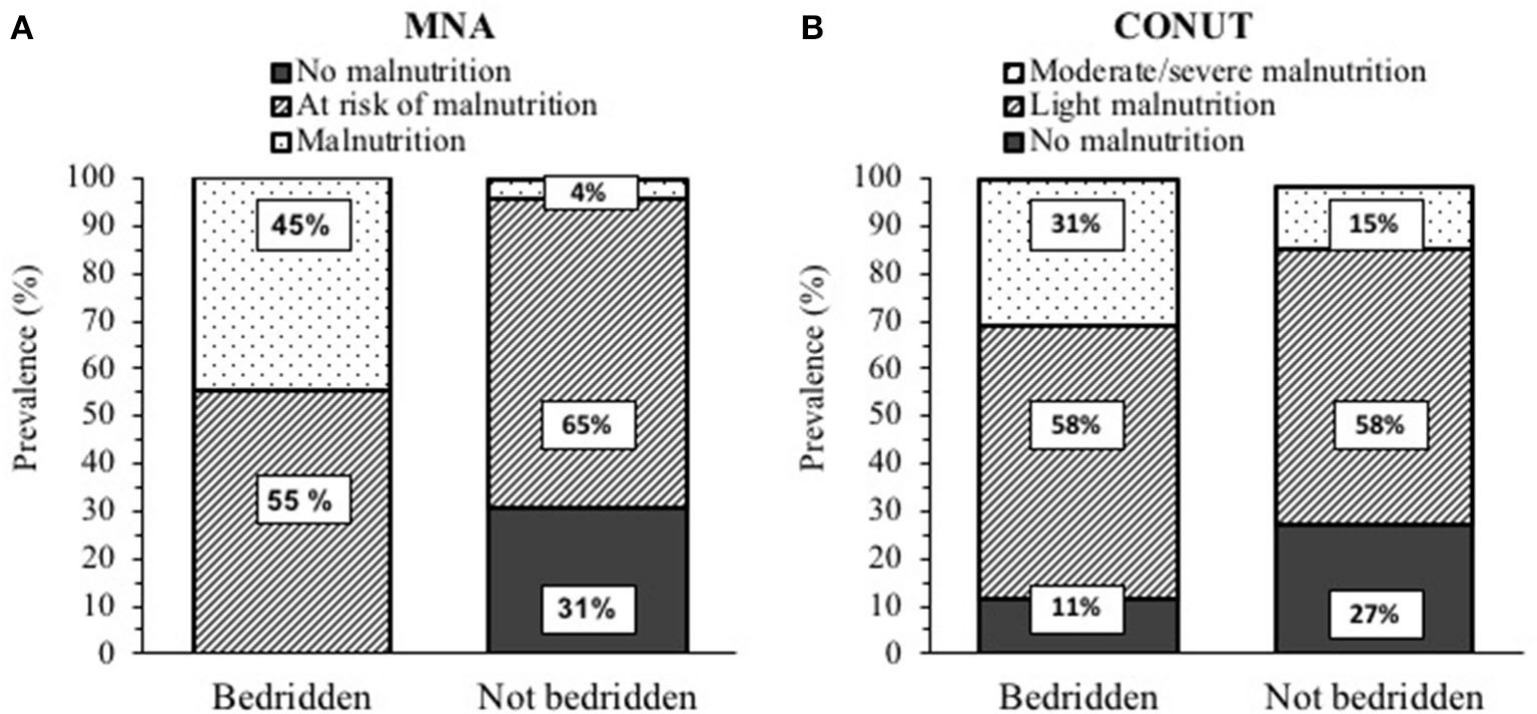
Prevalence of malnutrition in post-acute COVID-19 patients (53 bedridden and 91 not bedridden) according to: **(A)** Mini Nutritional Assessment (MNA). and **(B)** Controlling nutritional status (CONUT). The *p* < 0.01 for differences in frequencies between the bedridden and not bedridden patients for both MNA and CONUT.

Bedridden patients were older and had lower weight and BMI compared to the not-bedridden ones (Table 3), but a higher prevalence of low FFMI (16/53 *vs*. 8/91 patients, 30 *vs*. 9%) and SMI (32/53 *vs*. 12/91 patients, 60 *vs*. 13%). As shown in Figures 3A,B, they were more frequently malnourished according to MNA and CONUT. Even after controlling for sex, age, and FFM, PhA was lower and IRs were greater in bedridden *vs*. the other patients (*p* < 0.01, data not shown). Similarly, HGS was lower by 33% in patients confined to bed (*p* < 0.001).

**Table 3:** Body composition and muscle strength comparison in bedridden *vs*. not bedridden post-acute COVID-19 patients stratified by sex.

	**Males**		**Females**	
	**Bedridden**	**Not bedridden**	**Bedridden**	**Not bedridden**
	**(*n* = 32)**	**(*n* = 63)**	**(*n* = 21)**	**(*n* = 28)**
Age (age)	67.7 ± 11.5	62.6 ± 10.0^*^	69.1 ± 8.7	63.0 ± 11.5^*^
Weight (kg)	77.4 ± 17.5	85.6 ± 17.4^*^	74.0 ± 11.4	78.5 ± 19.5
Stature (cm)	171.5 ± 8.5	172.1 ± 7.8	159.1 ± 6.9	159.2 ± 6.0
Body mass index (kg/m^2^)	26.2 ± 4.9	28.8 ± 5.1^*^	29.3. ± 4.7	31.0 ± 7.9
Fat-free mass (kg)	50.8 ± 8.0	57.1 ± 7.4^**^	41.8 ± 5.2	44.4 ± 6.6
Skeletal mass (kg)	23.2 ± 4.6	27.7. ± 4.2^***^	16.3 ± 4.1	18.6 ± 3.1^*^
Percentage body fat (%)	30.5 ± 9.2	32.2 ± 6.1	41.5 ± 7.7	42.2 ± 6.0
Fat-free mass index (kg/m^2^)	17.6 ± 1.4	19.3 ± 1.8^***^	16.5 ± 2.0	17.6 ± 2.5
Skeletal mass index (kg/m^2^)	7.8 ± 1.1	9.3 ± 1.1^***^	6.5 ± 1.7	7.3 ± 1.1^*^
PhA at 50 kHz (degrees)	3.18 ± 0.93	4.56 ± 0.92^***^	2.95 ± 0.62	4.04 ± 1.03^***^
IR Z 100 kHz/Z 5 kHz	0.897 ± 0.029	0.858 ± 0.030^***^	0.905 ± 0.022	0.878 ± 0.028^***^
IR Z 200 kHz/Z 5 kHz	0.844 ± 0.034	0.800 ± 0.035^***^	0.855 ± 0.029	0.820 ± 0.033^***^
Handgrip strength (kg)	18.8 ± 5.7	31.5 ± 7.0^***^	12.0 ± 4.7	18.7 ± 6.0^***^

*Data are expressed as mean ± SD*.

*The significance was calculated between sexes within each group (bedridden vs. not bedridden)*.

* *p < 0.05*;

** *p < 0.000*;

*** *p < 0.001*.

*COVID-19, coronavirus disease-2019; PhA, phase angle; IR, impedance ratio; handgrip strength, maximum value considering both arms*.

## Discussion

The present study combined the evaluation of nutritional status, BC, and muscle strength in a cohort of post-acute COVID-19 patients referred to a rehabilitation center after hospital discharge. A high prevalence of malnutrition was observed. Patients exhibited marked abnormalities of raw BIA variables and HGS, which were more prevalent in those bedridden or malnourished, and when FFM or SM was low.

The poor nutritional status in COVID-19 patients may be due to multiple factors such as inflammation, hyper-catabolism, increased work of respiratory muscles, *etc* (2–4); so far, using different tools and criteria, the risk of malnutrition has been estimated between 39 and 86%, and the prevalence of malnutrition from 5 to 69% (2, 28). In the patients we studied, the nutritional assessment was performed with two different tools, MNA and CONUT, which have been already related to hospitalization, adverse outcomes, and mortality in COVID-19 disease (29–36); For consistency within the sample, MNA was also applied in patients aged <60 years (27% of total). According to the long form of MNA, 62% of the patients were at risk of malnutrition and 18% were malnourished, in agreement with the data of Haray et al. (29) in ICU COVID-19 patients, but not with those of Kananen et al. (31), who used the MNA short form in elderly hospitalized COVID-19 patients. According to the CONUT score, moderate-severe malnutrition was detected in 21% of our patients, a prevalence lower than that previously observed in acute hospitalized patients (32, 36). Interestingly, 33% of the patients were identified as malnourished by one of the two tools, but only 6% by both, suggesting a low agreement between MNA and CONUT. Finally, the prevalence of malnutrition was related to the number of comorbidities only for MNA but not for CONUT (data not shown), in partial disagreement with previous studies (32, 36).

As for BC, while obesity and increased FM have frequently been associated with disease severity, length of hospital stay, ICU admission, and death (3, 20, 37–44), only a few studies have yielded data on muscle wasting, low FFM, and appendicular skeletal muscle in COVID-19 patients (4, 7, 8, 14); for instance, a low pectoralis muscle area was found to be a predictor of poor prognosis (45). According to the data of this study, low FFMI and low SMI (by BIA) were observed in 17% and 31% of the whole sample, respectively (no previous evidence is available in the literature), being more common in malnourished patients as identified by MNA. This is not surprising in light of the expected impact of a severe catabolic disease state on muscle mass.

Directly measured raw BIA variables may be considered as indexes of BCM and ECW/TBW ratio; the interest in assessing PhA and IR is, therefore, justified by the idea to get additional information in the clinical setting on the inherent characteristics and composition of FFM (46, 47). Low PhA has already been observed in hospitalized COVID-19 patients and related to disease severity and prognosis (10, 48, 49), with little evidence in post-acute COVID-19 (8, 12) and no data on IRs, while abnormalities in both PhA and IRs have already been reported by our group in malnourished patients with chronic obstructive pulmonary disease (COPD) (50).

The phase angle or IRs widely varied in our patients but indeed clearly differed from values reported in the general population (51, 52) and also in well-nourished COPD patients (53). Concerning patients' characteristics, we found a significant change of either PhA or IRs with age, which is in line with what is observed in the general population (51, 52). Furthermore, low PhA and higher IRs were more common in patients with low SMI, in the malnourished ones (as identified by MNA or CONUT), and in those with higher CRP values, this latter finding is in agreement with previous evidence in the acute phase of the disease (11).

As for muscle function, it is worth recalling that in critical ICU COVID-19 patients, muscle wasting and decreased muscle strength occurred early and rapidly (14) and persisted at 3 months from discharge (15), with similar observations also in mild-to-moderate disease (16). COVID-19 can accelerate the aging process of institutionalized older adults (17), while lower HGS is an independent risk factor for disease severity (18, 19), hospital stay (20), and mortality (21).

In the post-acute COVID-19 patients we studied, HGS was much greater in males compared to females, as already reported (8, 54), and lower in older patients. Using the EWGSOP 2019 criteria, the percentage of dynapenic patients was 65% in males and 47% in females; this prevalence is much greater than the one determined by our group in patients with COPD (unpublished data) or idiopathic pulmonary fibrosis (55). HGS was also more strongly correlated with PhA and IRs than BC, confirming that raw BIA variables are potential markers of muscle function. Furthermore, HGS was lower in malnourished patients than in the remaining ones only when MNA was used (not with CONUT), highlighting a difference between these two tools that deserve further investigation. Of note, the notion that acute inflammation may impact muscle strength was confirmed by the inverse correlation between HGS and CRP.

Finally, concerning clinical features, while BC was not significantly different in hypertensive patients, the diabetic ones exhibited lower PhA and HGS, and higher IRs. More interestingly, the bedridden state was clearly associated with a poor nutritional status, with a higher prevalence of malnutrition and low FFMI or SMI values, as well as lower PhA and HGS, and higher IRs. This is likely due to the complex effects of physical inactivity on muscle, for instance, reduced stimulation of muscle protein synthesis by amino acids.

The limitations of this single-center study should be acknowledged. First, as patients were evaluated at admission to a rehabilitation unit, they were not representative of all hospitalized COVID-19 patients at discharge. Comparison data for the hospitalization period were not available because the efforts needed to manage COVID-19 along with restrictions aimed to contain infection spreading made it difficult to carry out specific studies during the active phase of the disease. BC was evaluated by a field method and not by a criterion method such as dual energy X-ray absorptiometry (DXA), which indeed cannot be easily applied in such a context. The effects of supine position on measuring HGS cannot be easily weighed; indeed, HGS was determined with the elbow straight and fully extended in all patients.

## Conclusion

We provide evidence of a high prevalence of malnutrition, marked abnormalities of BC and raw BIA variables, and low HGS in a cohort of post-acute COVID-19 patients, referred to a rehabilitation center. The poor nutritional status was more prevalent in bedridden patients. Further studies will help to identify factors associated with malnutrition, to define an appropriate approach to evaluate muscle composition and function and to tailor such information for nutrition care processes and rehabilitation strategies.

## Data Availability Statement

The original contributions presented in the study are included in the article/Supplementary Material, further inquiries can be directed to the corresponding author.

## Ethics Statement

The studies involving human participants were reviewed and approved by Azienda Ospedaliera dei Colli (AOC), Naples, Italy. The patients/participants provided their written informed consent to participate in this study.

## Author Contributions

FB, LS, AS, and MB contributed to the conception and design of the study. FB, GB, AS, and MB contributed to patients recruitment. FB, BC, AS, GB, LC, and PA contributed to the study test performance and data collection. LS and MB authored the manuscript. All authors contributed to data analysis and interpretation, provided a critical review of the manuscript, and approved it.

## Conflict of Interest

The authors declare that the research was conducted in the absence of any commercial or financial relationships that could be construed as a potential conflict of interest.

## Publisher's Note

All claims expressed in this article are solely those of the authors and do not necessarily represent those of their affiliated organizations, or those of the publisher, the editors and the reviewers. Any product that may be evaluated in this article, or claim that may be made by its manufacturer, is not guaranteed or endorsed by the publisher.

## Abbreviations

COVID-19: Coronavirus disease 2019
BC: body composition
FFM: fat-free mass
SM: skeletal muscle mass
MNA: mini-nutritional assessment
CONUT: ontrolling nutritional status
BIA: bioelectrical impedance analysis
PhA: phase angle
IR: impedance ratio
HGS: handgrip strength
SARS-CoV-2: severe acute respiratory syndrome-Coronavirus-2
ICU: intensive care unit
BCM: body cell mass
ECW: extracellular water
TBW: total body water
RT-PCR: reverse transcriptase polymerase chain reaction
BMI: body mass index
Impedance: Z
D: dominant
ND: non dominant
FFMI: fat-free mass index
SMI: skeletal muscle mass index
SD: standard deviation
IQR: interquartile range
SE: standard error
GLM: general linear model
CRP: C-reactive protein
COPD: chronic obstructive lung disease
DXA: dual energy X-ray absorptiometry.
