# Supplementary material for: Poor Nutritional Status and Dynapenia Are Highly Prevalent in Post-Acute COVID-19
Source: Front Nutr. 2022 Jun 3;9:888485. doi: 10.3389/fnut.2022.888485 (PMC9205211; doi:10.3389/fnut.2022.888485)
Supplement: Supplementary file 1 [file Table_1.docx]

Supplementary Table 1. Phase angle and IR Z200 kHz/Z 5 kHz according to MNA or CONUT categories in 144 post-acute COVID-19 patients

|  | **MNA categories** | | | | | |
| --- | --- | --- | --- | --- | --- | --- |
|  | No malnutrition  (n=29) | | At risk of malnutrition  (n=89) | | Malnutrition  (n=26) | |
| **PhA at 50 kHz (degrees)** | 4.72 | ±1.00 | 3.92 | ±1.06 | 3.05 | ±0.80 |
| **IR Z 200 kHz/Z 5 kHz** | 0.816 | ±0.032 | 0.834 | ±0.041 | 0.860 | ±0.033 |

|  | **CONUT categories** | | | | | |
| --- | --- | --- | --- | --- | --- | --- |
|  | No malnutrition  (n=30) | | Light malnutrition  (n=83) | | Moderate/severe malnutrition  (n=31) | |
| **PhA at 50 kHz (degrees)** | 4.13 | ±1.00 | 3.74 | ±1.13 | 3.82 | ±0.82 |
| **IR Z 200 kHz/Z 5 kHz** | 0.829 | ±0.034 | 0.841 | ±0.040 | 0.840 | ±0.029 |

Data are expressed as mean±standard deviation (SD)

All the pairwise differences between groups were statistically significant (p<0.001)

COVID-19= Coronavirus disease-2019; PhA= phase angle, IR= impedance ratio
